# Diagnosis and Management of Bladder Dysfunction in Neurologically Normal Children

**DOI:** 10.3389/fped.2019.00298

**Published:** 2019-07-25

**Authors:** Mirgon Fuentes, Juliana Magalhães, Ubirajara Barroso

**Affiliations:** ^1^Center of Urinary Disorders in Children (CEDIMI), Bahiana School of Medicine and Federal University of Bahia, Salvador, Brazil; ^2^Aliança Hospital, Salvador, Brazil

**Keywords:** incontinence, lower urinary tract dysfunction, overactive bladder, children, constipation, electrical nerve stimulation, neuromodulation

## Abstract

Normal bladder and urethral sphincter development as well as neural/volitional control over bladder-sphincter function are essential steps for regular lower urinary tract function. These maturational sequences are clinically evident by the age of 5 years. However, in 17–22% of children, symptoms persist beyond that age, characterizing lower urinary tract dysfunction (LUTD). The clinical spectrum is wide and includes overactive bladder, voiding postponement, underactive bladder, infrequent voiding, extraordinary daytime only urinary frequency, vaginal reflux, bladder neck dysfunction, and giggle incontinence. LUTD may lead to vesicoureteral reflux and recurrent urinary tract infections, increasing the likelihood of renal scarring. LUTD is often associated with constipation and emotional/behavioral disorders such as anxiety, depression, aggressiveness, and social isolation, making diagnosis, and treatment imperative. Diagnosis of LUTD is essentially based on clinical history, investigation of bladder storage, voiding symptoms (urinary frequency, daytime incontinence, enuresis, urgency) and constipation. Dysfunctional Voiding Score System (DVSS) is a helpful tool. Physical examination focuses on the abdomen to investigate a distended bladder or palpable fecal mass, the lumbosacral spine, and reflex testing. Bladder diaries are important for recording urinary frequency and water balance, while uroflowmetry is used to assess voided volume, maximum flow, and curve patterns. Bladder ultrasonography to measure post-void residual urine volume and urodynamics are used as supplemental tests. Current first line treatment is urotherapy, a combination of behavioral measures to avoid postponing micturition, and a restricted diet for at least 2 months. Anticholinergics, β3 agonists and neuromodulation are alternative therapies to manage refractory overactive bladder. Cure rates, at around 40%, are considered satisfactory, with daytime symptoms improving in 32% of cases. Furthermore, children who are also constipated need treatment, preferentially with polyethylene glycol at doses of 1–1.5 g/kg in the 1st 3 days and 0.25–0.5 g/kg thereafter until the 2-month period of behavioral therapy is complete. If urotherapy fails in cases of dysfunctional voiding, the next step is biofeedback to teach the child how to relax the external urethral sphincter during micturition. Success rate is around 80%. Children with underactive bladder usually need a combination of clean intermittent catheterization, alpha-blockers, biofeedback and neuromodulation; however, cure rates are uncertain.

## Introduction

The physiological function of the bladder and lower urinary tract develops as children grow. Socially acceptable conditions are achieved progressively, with voiding control being reached at around 5 years of age. When lower urinary tract symptoms (LUTS) persist beyond that age, the condition is referred to as lower urinary tract dysfunction (LUTD). This condition has been reported to affect 17–22% of children (1–3).

Children with LUTD are more likely to develop urinary disorders such as vesicoureteral reflux and recurrent urinary tract infection (UTI). These conditions increase the likelihood of renal scarring (4), which, over the long term, may progress to kidney failure (5–7). Children and adolescents with LUTD may also express emotional and behavioral disorders such as anxiety, depression, aggressiveness, and social isolation. It's unknown which one is the cause and the consequence (8); the pathological condition underlying this association is a complex problem. In a trial, 29.4% of patients with LUTD were diagnosed with concomitant psychiatric disorders, mainly attention deficient hyperactivity disorder (8). In addition, they run an increased risk of experiencing bullying (4, 9).

LUTD may be associated with other conditions, the most common being constipation. Children with constipation have been reported to be 6.8 times more likely to have LUTD compared to children with normal bowel function (10). A probable explanation for this phenomenon is that the bowel and bladder share afferent nerves, similar neural centers, and a common embryological origin. When constipation and LUTD are both present, the condition is referred to as bladder and bowel dysfunction (BBD) (11).

The term LUTD refers to different clinical characteristics depending on the physiopathology and symptomatology involved. According to the International Children's Continence Society (ICCS), LUTD encompasses the following range of symptoms: overactive bladder (OAB), voiding postponement, underactive bladder, infrequent voiding, extraordinary daytime only urinary frequency (EDOUF), vaginal reflux, bladder neck dysfunction, and giggle incontinence.

This review article dedicates to describe the diagnosis and treatment of LUTD according to the most recent publications.

## Diagnosis

**Figure 1:**
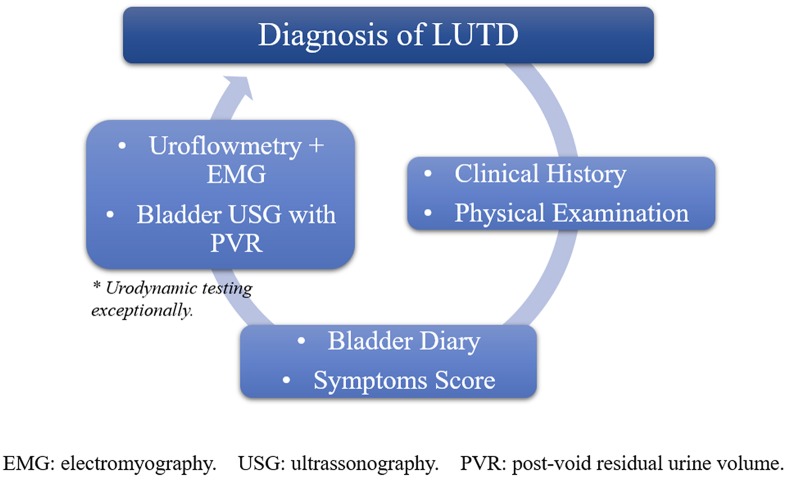
Illustration showing a representation of diagnostic tools for LUTD.

Diagnosis of LUTD is essentially based on clinical history, physical examination, bladder diary (BD), symptoms score such as the Dysfunctional Voiding Score System (DVSS), associated with uroflowmetry and bladder ultrasonography to measure post-void residual urine volume (Figure 1).

### Clinical History

The diagnosis of LUTD is essentially based on a thorough evaluation of the patient's clinical history. Anamnesis will focus on the patient's medical and surgical history (12), including any previous medications used to treat the current pathology and any other non-pharmacological treatments such as behavioral therapy or physiotherapy. Bladder storage, voiding symptoms and constipation should be investigated (6, 13). Urinary frequency, hesitancy, daytime incontinence, enuresis, urgency, urge incontinence, pelvic pain, and urine flow patterns are also relevant. None of these symptoms is expected to be present in children over 5 years of age; otherwise, the child should be managed as having LUTD.

To avoid involuntary urine loss, children often perform holding maneuvers. In girls, these usually consist of crossing their legs, with one knee over the other, pressing their genitals, walking on tiptoe and squatting. It has been reported that 73% of children with LUTD perform holding maneuvers (14).

The presence of constipation is evaluated based on reported symptoms such as excessive straining during defecation, hard stools, a feeling of incomplete evacuation or anorectal blockage, the use of manual evacuation maneuvers to facilitate defecation or the occurrence of fewer than three spontaneous evacuations per week (10). Patients will be asked about the characteristics of their stools. Recognizing BBD is crucial, since constipation needs to be treated together with voiding issues. Treating this disorder usually reduces urinary symptoms and decreases the likelihood of developing a UTI (15, 16).

Mothers of children with LUTD have also been shown to be more likely to have voiding symptoms. If constipation is included, this likelihood is even greater (17).

Furthermore, questions should be asked regarding the child's psychological development, particularly with respect to stressful events that may have occurred in the life of these children, the presence of anxiety, excessive shyness, aggressiveness, oppositional behavior, attention deficit/hyperactivity disorder, etc. (8). If an emotional disorder is suspected, the child should be referred for psychological evaluation (8).

### Physical Examination

Physical examination focuses on the possibility of a distended bladder and the presence of a palpable fecal mass in the abdomen or possible fecal impaction in the rectal ampulla (18). In this clinic, rectal examination is not routinely performed so as to avoid causing discomfort to the children. A complete examination of the lumbosacral spine is performed to investigate for defects suggestive of spina bifida and the cremasteric and anal sphincter reflexes are tested (18).

### Bladder Diary (BD)

The bladder diary is used to record urine output and frequency as well as fluid intake and any occurrence of urgency or involuntary urine loss. Recently, a bladder diary maintained over two, not necessarily consecutive, days has been shown to provide data that are similar to those obtained over a 3-day period (19). The color of the urine is also routinely evaluated to assess the patient's state of hydration. A possible bias must be considered, on account of BD use may lead to a bladder training effect and nights during recording have evidenced relevant variations in the frequency of nocturnal voids. Despite that, it continues to be one of the most important and simplest tools to access LUTS, setting more confidence to the diagnosis commonly overestimated by DVSS (20).

### Symptoms Score

Specific questionnaires have been developed for use in the investigation of LUTS, including the Dysfunctional Voiding Score System (DVSS), the Dysfunctional Voiding and Incontinence Score System (DVISS), and the Incontinence Symptoms Index-Pediatric (ISI-P) (6). These scores have little relevance in clinical practice; however, they are important tools in clinical investigation, particularly with respect to standardizing the terms used and as a guide for professionals with less experience. They are complementary tools to BD, in order to evaluate not only the actual voiding but also the self-reported behavior, a direct reflection of how LUTD impacts (20).

**Figure 2:**
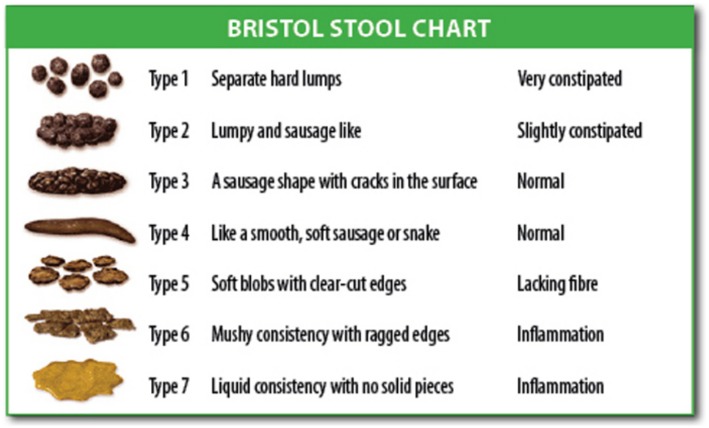
Illustration showing the Bristol Stool Chart. (Source: The Organic Dietitian). Source: Wikipedia Commons. Bristol Stool Scale. [online publication] Salvador, 2019. Wikipedia Commons October 22, 2015 (accessed on July 2, 2019). Available at: https://commons.wikimedia.org/wiki/File:Bristol_stool_chart.svg.

Due to the significant association between constipation and LUTD, this condition should also be assessed through the use of questionnaires such as the Rome IV diagnostic criteria and the Bristol stool scale (Figure 2). We believe these scores to be extremely important, since this pathology may fail to be diagnosed if incorrectly evaluated.

### Biomarkers

Nerve growth factor (NGF) and brain derived neurotrophic factor (BDNF) are messengers emitted from the uroepithelial bladder smooth muscle aiming to differentiate neuronal cell in peripheral nervous system. These biomarkers are believed to be increased in the urine of patients with OAB (21). Studies have already observed decreased urine levels during treatment as well as an increase after quitting the treatment, inciting questions about the biomarker's real role in the pathophysiology. So far, results show they could not predict who would benefit from the treatment with antimuscarinics, but more robust studies are yet in course (21).

## Non-Invasive Additional Evaluation

Our group has previously shown that, although clinical history is sufficient to enable a diagnosis of LUTD to be reached, it is not that specific for the identification of abnormalities in the emptying phase, such as dysfunctional voiding (22). Therefore, once the patient's clinical history has been thoroughly investigated, additional examinations may prove necessary. Urinalysis is one of the tests available, together with urine culture if a UTI is suspected. Samples for urinalysis are simple to collect, and in certain cases, can guide the investigation toward specific urinary tract pathologies (22).

### Uroflowmetry

Uroflowmetry is an indispensable test for children with LUTD. Simple to perform, non-invasive and fast, this test provides important data such as: voided volume, voiding time, maximum flow, curve pattern, and rate of flow. Uroflowmetry results showing a voided volume of at least 50 ml or 50% of the expected bladder capacity for age [(age in years + 1) ×30] are considered adequate (23, 24).

Uroflowmetry provides relevant data on lower urinary tract (LUT) function and on the possible etiology of LUTD. The type of curve pattern identified at uroflowmetry may suggest specific conditions. A bell-shaped curve is considered physiological. Conversely, a staccato-shaped pattern suggests dysfunctional voiding or bladder and bladder neck dysfunction, while a tower-shaped curve is indicative of OAB, a plateau is suggestive of a LUT obstruction and an interrupted curve is typical of an underactive bladder. Interpreting uroflowmetry curves can be subjective and conclusions may vary from one professional to another (23, 24).

The flow index (FI) is a new parameter that is now available and is reached by dividing the actual Qmax by the estimated Qmax. In boys, an FI <0.70, is indicative of a flat curve, while an FI of 0.71 to 1.25 suggests a bell-shaped curve, and values > 1.25 are typical of a tower-shaped curve. In girls, an FI < 0.68 strongly implies a flat curve, while values of 0.69 to 1.1 suggest a bell-shaped curve, and values > 1.1 suggest a tower-shaped curve (23, 24).

Uroflowmetry with electromyography (uroflow-EMG) is a non-invasive evaluation method that provides information on the functionality of the pelvic floor and external sphincter. The procedure consists of attaching electrodes in the perineal area at the 3 and 9 o'clock positions. Simultaneous use of electromyography during uroflowmetry adds relevant data during micturition, including coordination or lack of coordination in the relaxation of the sphincter. In addition, this method provides the lag time, i.e., the time required for the pelvic floor to relax once micturition is initiated, which should be between 2 and 6 seconds. The concept that the time the pelvic floor takes to relax immediately prior to detrusor contraction and the beginning of voiding is longer in patients with dysfunctional voiding is widely accepted (25). No pelvic floor activity is expected during voiding in patients with genuine OAB. Conversely, patients with dysfunctional voiding will have a tonic contraction of the sphincter during voiding (sharp peaks on the EMG) (26). This method is susceptible to artifacts, and the electromyographic activity does not always reveal sphincter activity. The effectiveness of electromyography for tower-shaped and bell-shaped curves has yet to be clarified (27).

### Bladder Ultrasonography and the Measurement of Post-Void Residual Urine Volume

**Figure 3:**
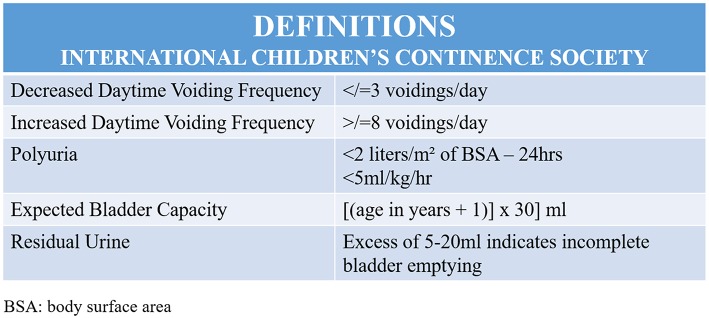
Illustration showing the International Children's Continence Society (ICCS) definitions.

For reliable data to be obtained, bladder volume should be between 50 and 115% of the expected capacity for age. In addition, post-void residual urine volume should be measured in the 1st 5 min after micturition and, in ideal conditions, <1 min after micturition. Post-void residual urine volume <20 ml on more than one occasion is considered abnormal (Figure 3) (28). Bladder wall thickness, measured on an empty bladder, should be <3 mm. In constipated children, measuring the rectal diameter is also helpful, with measurements >3 cm being considered clinically significant. Dynamic bladder ultrasonography has been reported as being useful in evaluating pelvic floor contraction when involuntary contractions are present in cases of OAB. However, the clinical relevance of these findings has yet to be established (29).

### Urodynamic Testing

Urodynamic testing in children with LUTD and no neurological abnormalities is only recommended when treatment has persistently failed, in cases of underactive bladder, or when there is significant bilateral dilation of the upper urinary tract. In this situation, video urodynamics is a useful tool with which to identify vesicoureteral reflux and differentiate between bladder neck and bladder sphincter discoordination (28).

## Classification (ICCS)

### OAB

The presence of urinary urgency is usually associated with daytime urinary incontinence and frequency.

### Voiding Postponement

Low daytime micturition frequency (≤ 3 times/day), but with no post-void residual urine. In such cases, the child usually performs holding maneuvers to postpone voiding.

### Dysfunctional Voiding

Discordant bladder and perineal activity, with or without post-void residual urine. The child may fail to relax the bladder neck during micturition.

### Underactive Bladder

Low voiding frequency during the day (≤ 3 times/day) associated with a large amount of post-void residual urine. Detrusor function is abnormal at urodynamic testing. This is the only situation in which urodynamic testing is normally recommended as part of the initial workup.

### Giggle Incontinence

Incontinence when the child laughs.

### Vaginal Reflux

This occurs due to the position adopted by some girls, particularly overweight girls, while voiding. As the girl gets up, urine dribbles from the vagina.

### EDOUF

A sudden increase in daytime voiding frequency unrelated to either urgency or incontinence. Generally associated with anxiety and stressful events.

All these conditions are frequently intertwined. For instance, children with dysfunctional voiding often have symptoms of OAB.

## Treatment

**Figure 4:**
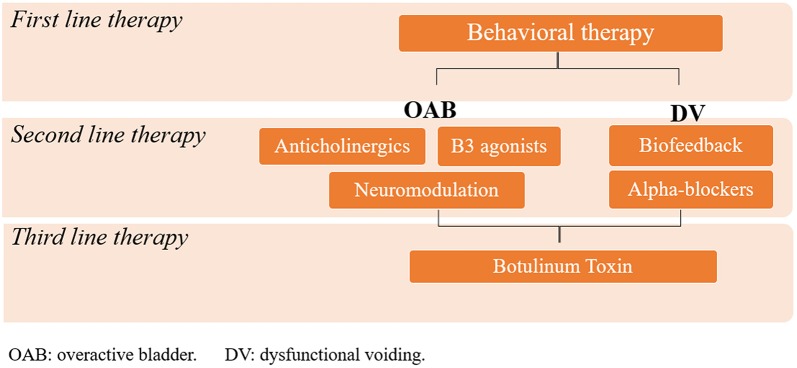
Illustration showing an schematic representation of lines of treatment of LUTD.

First line therapy is aimed at improving voiding habits in children with LUTD (Figure 4).

### Behavioral Therapy (Urotherapy)

**Figure 5:**
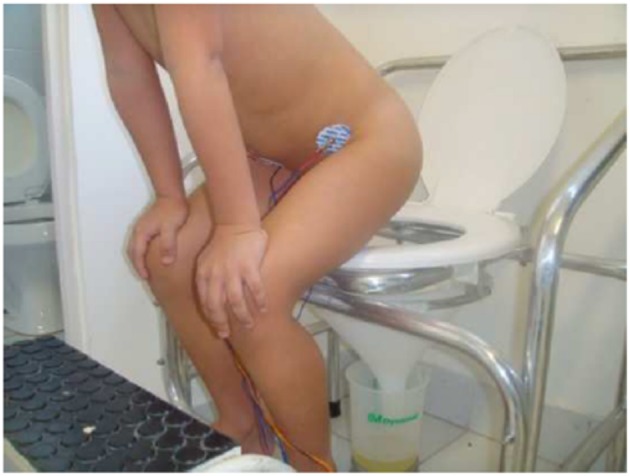
Illustration showing a wrong voiding posture, using pelvic floor muscles. Written informed consent was obtained from this patient and his/her legal guardians authorizing publication of this image.

**Figure 6:**
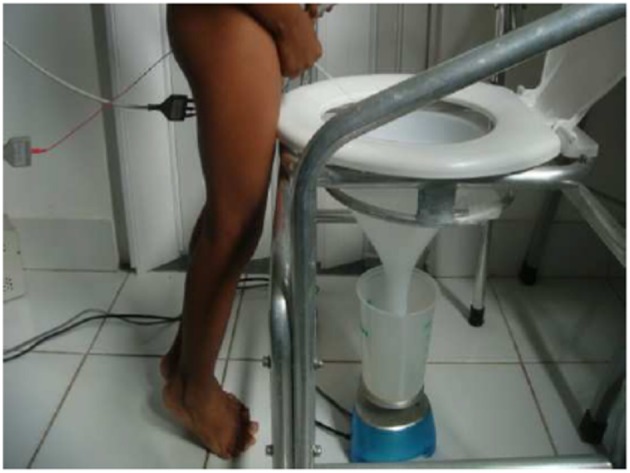
Illustration demonstrantes another incorrect voiding posture. The tip toe position activates pelvic floor muscles. Written informed consent was obtained from this patient and his/her legal guardians authorizing publication of this image.

This form of treatment consists of training children and adolescents to void every 3 h and whenever they feel the need to urinate or when they perform holding maneuvers. They are instructed to ensure that their fluid intake during the day is adequate and to restrict their consumption of caffeine, chocolate and citrus fruits. The position adopted during micturition and defecation also merits attention. Both feet should be resting on a flat surface and the coordination between the relaxation of the pelvic muscle and the bladder contraction should be closely observed (Figures 5, 6) (30). Urotherapy must be continued for at least 2 months, with reevaluation required at the end of this period. Nowadays, there are mobile applications aimed at helping patients during behavioral therapy. Furthermore, for children who are also constipated, treatment with polyethylene glycol should be initiated, at doses ranging from 1 to 1.5 g/kg in the 1st 3 days and 0.25–0.5 g/kg thereafter until the 2-month period of behavioral therapy is complete (4).

This modality of treatment works better for patients who tend to postpone voiding, with cure rates of around 40% and an improvement rate of 32% in daytime symptoms (31).

Once this initial line of treatment is complete, if symptoms persist, individualized management is required, with the type of treatment depending on the kind of dysfunctional voiding present. For those with genuine OAB in whom initial behavioral therapy failed, anticholinergics are the first line of choice for pharmacological therapy (32), and neuromodulation (transcutaneous parasacral or posterior tibial nerve stimulation) is the best non-pharmacological option (14). However, OAB often presents in association with other types of LUTD, such as voiding postponement and dysfunctional voiding. The approaches used and the different forms of management for each case are described and discussed below.

### Second Line and Additional Therapies

When urotherapy fails, other forms of treatment are required. Different treatment options should be considered for the different conditions.

## OAB

OAB can be managed either by medication, by neuromodulation, or by a combination of both.

### Anticholinergics and β3 Agonists

These drugs are better suited for the treatment of genuine OAB, when patients do not postpone micturition and void correctly. Antimuscarinics are widely used; however, treatment is often discontinued due to a lack of effect or bothersome side effects. These drugs act by antagonizing the response to acetylcholine and other parasympathomimetic substances that are mediated through activation of the muscarinic receptors (principally M2 and M3) in the bladder (33). The beta-3 agonist, mirabegron, was introduced in 2012 as a new drug for the treatment of OAB (33).

In one analysis, oxybutynin, and solifenacin were found to be the most cost-effective antimuscarinics (34).

Oxybutynin is the most commonly prescribed anticholinergic. The recommended dosage is 0.2 to 0.6 mg/kg (with a maximum dose of 15 mg/day) and its effectiveness has been reported as around 30–40% (35). Although oxybutynin has been the most commonly prescribed medication for OAB in children for years, no randomized clinical trial has yet been conducted to compare this drug with placebo. Tolterodine, available in tablets of 1 and 2 mg, can also be used for the treatment of OAB. In one systematic review, the dose of tolterodine used in trials ranged from 0.8 to 8 mg/day (1). Overall, the success rate was comparable to that of oxybutynin (1). A randomized clinical trial compared tolterodine and placebo and failed to show any difference in outcome between the groups (2).

The antimuscarinic solifenacin has a long half-life. Although still off label, the drug has been used at a dose of 5 mg once a day. One study reported a complete response rate of 53% (3). In another prospective multicenter study, treatment with solifenacin resulted in improvement, as shown by the difference in maximum voided volume between baseline and the end of treatment, and in a decrease in voiding frequency (4). However, in one randomized clinical trial the rate of resolution of LUTS with solifenacin was similar to that found with placebo (5). In patients with refractory OAB, combination therapy is an option in more refractory cases. However, treatment with the combined medication was discontinued in 22% of patients due to adverse events and in 54% due to a lack of efficacy (36).

The side effects of antimuscarinics include constipation, dry mouth, heat intolerance and, more rarely, mental confusion increasing the discontinuation rate with oxybutynin to 32% (37). The prevalence of these side effects has been described as follows: of the 53% of patients who reported a side effect, 29% reported dry mouth, 19% pruritus, 8% vertigo, 8% constipation, 6% headache, and 2% another side effect (37).

Mirabegron is a selective beta-3 adrenoceptor agonist with a different pharmacologic profile and mechanism of action to those of antimuscarinics. Bladder relaxation is obtained through activation of the beta-3 adrenoceptor and the subsequent activation of adenylyl cyclase (36). In adults, Mirabegron has been shown to be effective for the treatment of OAB, with few side effects; however, it is off label for children (38). One study reported a complete response rate in 22% of 58 children, and almost all the patients experienced some improvement in the severity of the symptoms of incontinence (6). In one study, mirabegron, and solifenacin were both shown to improve OAB symptoms, with no statistically significant difference between the two treatments. Both drugs were well-tolerated (38). Combination treatment with antimuscarinics and mirabegron may represent a promising option for patients who fail to respond to monotherapy (39). Although, the aim of the investigation is still to deduce how durable the effect of Mirabegron is in successfully-treated overactive bladder patients. A multicenter study involving adult and elderly participants concluded that most patients who discontinued treatment (69%) could only do so temporarily, considering a worsening of symptoms rapidly occurred, in an average of 48 days (increased number of frequency, urgency, and nycturia) (40).

In summary, anticholinergics may prove effective for children with OAB (35). Some randomized clinical trials have failed to find any difference between these medications and placebo, probably because of the high effectiveness of urotherapy in those studies (35–37). This means that the sample studied consisted of patients with voiding postponement plus OAB, which is the condition that responds best to behavioral therapy. The most common problems with antimuscarinics are the high rate of side effects (including the possibility of these drugs crossing the blood-brain barrier, thus causing mental confusion), the fact that constipation may worsen, and that treatment time is unknown (37).

### Neuromodulation

There are different modalities of neuromodulation that can be useful for the treatment of OAB, including parasacral transcutaneous electrical nerve stimulation (TENS), posterior tibial nerve stimulation (PTNS) and sacral implants (41).

Parasacral TENS has traditionally been performed with electrodes placed bilaterally in the region of S3, with different energy parameters and periodicity. The usual current frequency is 10 HZ (41). Pulse widths have varied from 100 to 700 microseconds (42). The intensity of the current is increased according to the patient's tolerance level.

With parasacral TENS, there is complete resolution of the symptoms of OAB in 63% to 73% of cases (43). The outcome with this treatment proved better than with sham in two randomized clinical trials (44, 45). One study reported a higher success rate for TENS + urotherapy compared to urotherapy alone (67 vs. 43%) in children with OAB who had failed to respond to previous treatments; however, the difference was not statistically significant, perhaps because the sample was underpowered for this evaluation, since the success rate with urotherapy is high. However, for patients who had undergone no previous treatment, the success rate was 71% in the TENS + urotherapy group and 48% in the urotherapy alone group (*p* = 0.05). In a study conducted by our research group, the cure rate with TENS for cases of OAB in children was 73%, with a recurrence rate of 10% over a follow-up period of at least 2 years (8).

TENS was compared with oxybutynin in a randomized clinical trial and the outcome was shown to be similar insofar as the improvement of symptoms was concerned. However, about half the patients randomized to oxybutynin experienced side effects directly related to the drug. Moreover, only those randomized to TENS experienced an improvement in constipation, which was achieved in 85% of cases (10, 11). The ability to manage BBD with just one treatment is another advantage of TENS (11).

When first described in children, the use of PTNS was shown to result in an improvement in 50% of the children with OAB, with 35% of the symptoms being completely resolved (12). Our group conducted a comparative study in which cure was achieved in 70% of children with OAB in the sacral TENS group compared to 9% in the PTNS group (46). It is possible that the distance from the posterior tibial nerve to S3 may be responsible for this difference in effectiveness (46).

**Figure 7:**
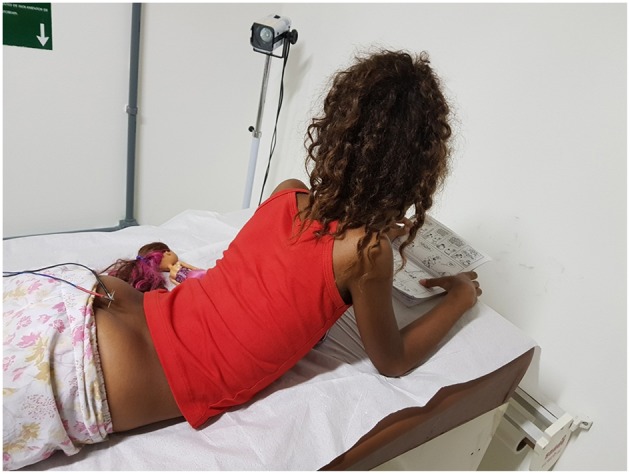
Illustration showing a child undergoing transcutaneous electrical nerve stimulation. Written informed consent was obtained from this patient and his/her legal guardians authorizing publication of this image.

With the idea of removing the impedance of the skin and getting closer to the S3 innervation, a new form of treatment was developed that consists of placing acupuncture needles at S3 (Figure 7). Sessions are performed once a week using the same electrical parameters established for parasacral TENS. In our pilot study of 17 cases, complete response to treatment was achieved in around 70% of cases, with minimal discomfort (46). Further studies are needed to test the efficacy of this method.

Sacral nerve modulation with an implantable pulse generator, i.e., the use of implantable sacral nerve stimulators, is currently undergoing evaluation in patients under 18 years of age. Groen et al. (47) reported an improvement in symptoms in 50% of treated children with OAB, all of whom were refractory to conventional pharmacological treatment (44). In another study, sacral nerve stimulation was found to improve the quality of life of children with LUTD (48).

In patients diagnosed with refractory overactive bladder, and especially those who do not wish to undergo invasive reconstruction surgery, the intravesical injection of Botulinum toxin A constitutes an innovative resource. The neurotoxin inhibits acetylcholine and adenosine triphosphate release from parasympathetic presynaptic nerve terminals, causing flaccid muscle paralysis. Previous trials evidence a complete response rate of 32–60% during a 6-month follow-up, and 44% of children were entirely dry at the end of 12 months (49). Studies conducted on adults found that the intravesical injections increased the risk of urinary retention by nine times. Nonetheless, this problem was minimally registered on pediatric population, and usually reversible (49). Many aspects of the technique are yet to be standardized, though. There is no data on whether to preserve trigon region or not during the injection, the number of injections varies widely, between 12 and 40, as well as the depth, since there isn't proof suggesting whether suburothelial injection is better than the intradetrusor or not (49).

## Dysfunctional Voiding

In cases of dysfunctional voiding, treatment is aimed at improving sphincter relaxation during micturition. Therefore, when urotherapy alone fails, the next step is biofeedback. The objective of this treatment is to teach the children how to relax the external urethral sphincter during micturition. The success rate with this procedure is around 80% (50, 51). Biofeedback sessions usually last 40 min and are performed once a week. Animated biofeedback helps children interact more with the treatment but does not change the final outcome. Patient or family motivation is essential in order to improve compliance. Children over 5 years of age engage better with biofeedback (41, 50).

When bladder neck dysfunction is suspected, alpha-blockers can be an option, although a randomized clinical trial with an admittedly small sample size failed to confirm the effectiveness of this treatment (52). Indications for alpha-blockers include a long EMG lag time at uroflowmetry, bladder neck dysfunction as shown at video urodynamics, and dysfunctional voiding patients who fail to respond to biofeedback to improve bladder neck relaxation (53). A few small studies have also reported good results in some patients using alpha-blockers combined with biofeedback to improve post-void residual urine (52, 54). The dose used is normally Doxazosin 1–2 mg daily (54).

For cases in which all treatments for dysfunctional voiding have failed, an external urethral sphincter injection of botulinum toxin may be an option. The usual doses range from 50 to 100 UI. This form of treatment normally results in improved post-void residual urine volume and urine flow 1 to 2 weeks after the injection. There is a possibility, however, that incontinence could develop secondary to the injection, with this usually resolving itself within 6 months of the procedure (15, 55). Moreover, the effectiveness of botulinum toxin for dysfunctional voiding has yet to be established.

Neuromodulation has also been used for dysfunctional voiding. Capitanucci et al. (55) reported that symptoms resolved completely with this method in around 85% of patients. Our group showed that this method can be used as salvage therapy for patients with dysfunctional voiding and OAB symptoms who fail to respond to biofeedback (56–58). According to the ICCS guidelines, antimuscarinics are indicated for patients with OAB symptoms and dysfunctional voiding who fail to respond to urotherapy. Nevertheless, although few studies have been published in the literature on this subject, in our view the bladder capacity of these patients tends to be large and they are likely to be constipated (13). Therefore, these patients are not good candidates for anticholinergics, since those drugs will increase bladder capacity even further and can worsen constipation (11).

## Underactive Bladder

There are no drugs currently on the market for the treatment of underactive bladder and a combination of measures is generally required. These measures include clean intermittent catheterization, alpha-blockers, biofeedback, and TENS. The therapeutic response to these measures, however, is uncertain. The implantation of sacral nerve modulators has been investigated (57, 59, 60); however, further studies are required.

## Antimicrobial Prophylaxis for Urinary Tract Infections

Continuous antimicrobial prophylaxis is generally recommended for children with vesicoureteral reflux, and according to large trials (RIVUR), and it is associated with a 2-fold reduction in risk to prevent recurrent urinary tract infections (61). Despite that, this practice has been questioned lately. Studies have noted that, since it requires regular therapeutic adherence, and it varies from 40–90% in this population, the risk of future renal scarring may not reduce. Plus, there are side effects of antibiotic exposure, remaining only the doubt as to whether this longstanding strategy is actually effective (61).

## Psychiatric Disorders

The interest in the association between psychiatric disorders and LUTD has been growing and generating and is an important field of research. Studies show that children with improvement in psychiatric disorders also had an improvement in their LUTD treatment compliance and a better overall outcome, but also that the treatment of voiding disorders may improve the psychiatric issues that originally required care (8). Therefore, it seems to be a good practice to apply a screening test for psychological problems and, if needed, refer these patients to appropriate care (8).

## Conclusions

In conclusion, neurologically normal children may present with symptoms of the lower urinary tract, which must be recognized in a timely manner by pediatricians so that treatment can be initiated as early as possible. With the multiple treatment modalities currently available, high cure rates can be achieved.

## Author Contributions

All three authors have made substantial contribution to the project, participating of all phases. MF wrote the first version, complemented by the work of JM. UB revised and performed modifications, lapidating the final work.

### Conflict of Interest Statement

The authors declare that the research was conducted in the absence of any commercial or financial relationships that could be construed as a potential conflict of interest.
